# Outcomes of transcatheter edge-to-edge mitral valve repair with percutaneous coronary intervention vs. surgical mitral valve repair with coronary artery bypass grafting

**DOI:** 10.3389/fcvm.2022.953875

**Published:** 2022-12-21

**Authors:** Xiqiang Wang, Yanpeng Ma, Jing Liu, Ting Wang, Ling Zhu, Xiude Fan, Qianwei Cui, Chengfeng Liu, Gongchang Guan, Junkui Wang, Shuo Pan, Zhongwei Liu, Yong Zhang

**Affiliations:** ^1^Department of Cardiovascular Medicine, Shaanxi Provincial People’s Hospital, Xi’an, Shaanxi, China; ^2^Department of Endocrinology, Shandong Provincial Hospital Affiliated to Shandong First Medical University, Jinan, Shandong, China

**Keywords:** transcatheter mitral valve repair, surgical mitral valve repair, functional mitral regurgitation, ischemic mitral regurgitation, National Inpatient Sample

## Abstract

**Aims:**

Patients with severe ischemic mitral regurgitation (IMR) may receive concurrent coronary artery bypass graft (CABG) with surgical mitral valve repair (SMVr) or percutaneous coronary stent implantation (PCI) with transcatheter edge-to-edge mitral valve repair (TMVr). However, there is no consensus on the management of severe IMR in this setting. We aimed to compare the outcomes of combined SMVr with CABG to concurrent TMVr with PCI among patients with IMR in the National Inpatient Sample (NIS) database.

**Methods and results:**

The National Inpatient Sample was queried for all patients diagnosed with IMR who underwent SMVr with CABG or TMVr with PCI during the years 2016–2018. Study outcomes included all-cause in-hospital mortality, periprocedural complications, and resources used. A total of 1,360 potentially eligible patients were included in the study. After 1:5 propensity score matching, 133 patients were classified in the SMVr + CABG group and 29 patients in the TMVr + PCI group. Adjusted mortality was higher in the TMVr + PCI group compared with the SMVr + CABG group (13.8% vs. 4.5%, *P* = 0.034). Perioperative complications were higher among patients who underwent SMVr + CABG including blood transfusions (29.3% vs. 6.9%, *P* = 0.01) and post-procedural cardiogenic shock (11.3% vs. 0%, *P* = 0.044). The cost of care was higher (USD$783548.80 vs. USD$331846.523, *P* = 0.001) and the length of stay was longer (17.9 vs. 15.44 days, *P* < 0.001) in the TMVr + PCI group. On multivariable analysis, age (OR, 1.039 [95% CI, 1.006–1.072]; *P* = 0.032), renal failure (OR, 3.465 [95% CI, 1.867–6.433]; *P* < 0.001), and liver disease (OR, 5.012 [95% CI, 2.578–9.686]; *P* < 0.001) were associated with in-hospital mortality.

**Conclusion:**

TMVr + PCI was associated with higher resource use and in-hospital mortality but with improved perioperative complications compared with SMVr + CABG.

## Introduction

The prevalence of ischemic etiology was reported to be 50% in patients with functional mitral regurgitation (FMR) (1). Previous studies had demonstrated that there were still plenty of patients with severe mitral regurgitation despite guideline-guided medical treatment (GDMT), cardiac resynchronization therapy, or coronary artery revascularization which were the first-line therapies for heart failure (HF) with ischemic mitral regurgitation (IMR) and used to improve the underlying left ventricular (LV) dysfunction (2). Surgical mitral valve repair (SMVr) for severe IMR in patients with LV systolic dysfunction has been demonstrated to improve symptoms and quality of life (3). However, a large number of patients are not referred for open-heart surgery (SMVr) because of their prohibitive surgical risk (4).

The COAPT randomized controlled trial (RCT) has shown that the use of transcatheter edge-to-edge mitral valve repair (TMVr) therapy is beneficial for the IMR (5), and TMVr is the only procedure that has gained widespread use in clinical practice. Although less effective than surgery in reducing MR, TMVr showed fewer perioperative adverse events and achieved a similar durable improvement in function (6, 7).

In the current clinical practice, patients with severe IMR and LV systolic dysfunction with suitable coronary targets affected by high-grade proximal stenosis may receive concurrent coronary artery bypass graft (CABG) with SMVr (8), and some patients may receive the concurrent TMVr and percutaneous coronary stent implantation (PCI). However, there is no consensus on the management of severe IMR in this setting. Given the limited literature on this topic, we aimed to investigate the in-hospital clinical outcomes of combined SMVr + CABG vs. concurrent TMVr + PCI in patients with IMR using a National Inpatient Sample (NIS) database.

## Materials and methods

### Study data

In this study, we used the NIS data from January 2016 to December 2018, which was developed by the Agency of Healthcare Research and Quality of the United States through a federal–state–industry partnership. The NIS database has more than 8 million inpatients and represents 20% of all hospital admissions in the United States. It is updated annually, thus we can use these data to analyze the disease trend over time (9). Because the NIS database is publicly available, we do not need to get the approval of the institutional review board or informed consent in our clinical study.

### Study design and data selection

**FIGURE 1:**
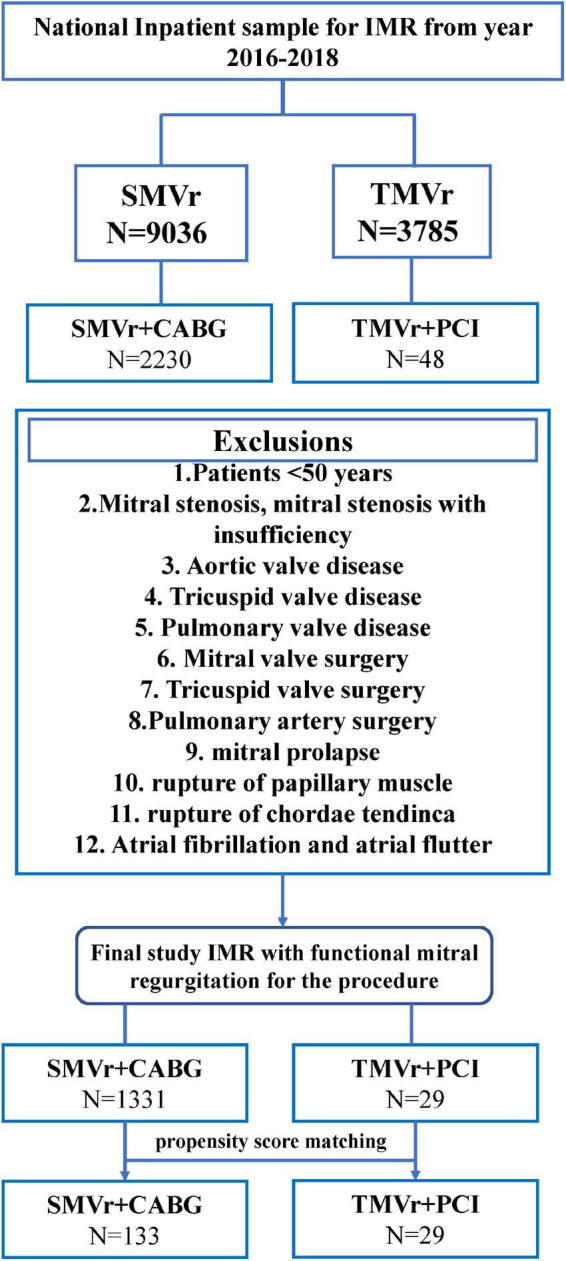
Flowchart of the study cohort. ICD-10-PCS indicates International Classification of Diseases, Tenth Revision, Procedure Coding System.

The International Classification of Diseases, Tenth Revision, Clinical Modification (ICD-10-CM) codes, and ICD-10-Procedure Coding System (PCS) codes were used to analyze these data. The NIS data from 2016 to 2018 were used in the present study (Supplementary Table 1). Consecutive patients with severe IMR, scheduled for concurrent CABG with TMVR or PCI with TMVr, were retrospectively analyzed in the NIS database. TMVR and CABG were performed as a single procedure, TMVr and PCI were performed as a staged procedure, and TMVr was performed after the PCI procedure. IMR with mitral valve insufficiency and without any other valvular disease was selected using the ICD-10-CM code. Patients who underwent TMVr or SMVr were selected by ICD-10-PCS codes, respectively. PCI or CABG were selected by ICD-10-PCS codes, and the periprocedural complications post the procedure were identified by the ICD-10-CM codes; the detailed ICD-10-CM codes and ICD-10-PCS codes are shown in Supplementary Table 1. Patients who were younger than 50 years old with mitral stenosis, mitral stenosis with insufficiency, aortic valve disease, tricuspid valve disease, pulmonary valve disease, mitral valve surgery, tricuspid valve surgery, pulmonary artery surgery, mitral prolapse, rupture of papillary muscle, rupture of chordae tendinae, and atrial functional mitral regurgitation (atrial flutter and atrial fibrillation) were excluded from our study. Propensity score matching was performed to adjust for confounding factors, resulting in 133 patients being assigned to SMVr + CABG and 29 patients assigned to TMVr + PCI groups, respectively. A flowchart of our patient selection criterion is shown in Figure 1.

### Study outcomes

The primary endpoints of our study were in-hospital mortality and periprocedural complications between the SMVR + CABG and TMVr + PCI groups. The secondary outcomes of interest were resources used and operative procedure-related trends over time, such as the length of hospital stay, total charges, and the age of patients who underwent SMVR + CABG and TMVr + PCI.

### Statistical analysis

Propensity score matching (PSM), a method to balance covariates in two groups by reducing the selection bias, was conducted to match patients who underwent SMVr + CABG to those who underwent TMVr + PCI. In our study, we included variables that may be associated with the outcome of patients with IMR of the NIS database in the propensity score model. Matching factors for 1:5 PSM include age, sex, hypertension, diabetes, heart failure, renal failure, and ICD implantation.

Pearson χ^2^ exact test was used for categorical variables, and the independent *t*-test was used for the continuous variables. The categorical variables and continuous variables were presented as frequency and median of standard deviations, respectively. Univariate and multivariate logistic regression analyses were performed to find the predictors of in-patient mortality, blood transfusion, and acute kidney injury. Model 1 indicates the univariate regression analysis; model 2 adjusted for SMVr + CABG, TMVr + PCI, age, female, race; model 3 adjusted for SMVr + CABG, TMVr + PCI, age, female, race, deficiency anemia, heart failure, renal failure, liver disease, chronic obstructive pulmonary disease, diabetes mellitus, hypertension, atrial fibrillation, peripheral vascular disease, cerebral infarction, coagulopathy, obesity, smoking, alcohol use, and hyperlipidemia. After SMVr + CABG and TMVr + PCI operations using relevant demographic and clinical variables were shown in Table 1. For all analyses, a two-sided *p*-value of <0.05 was considered statistically significant. Statistical analyses were performed using SPSS version 25 (IBM, Armonk, NY, USA) and R version 3.5 (version 3.6.3, R Core Team).

**TABLE 1:** Basic characteristics of the patients who underwent SMVr + CABG and TMVr + PCI (2016–2018).

	Unmatched groups			Propensity-matched groups		
**Characteristic**	**SMVr + CABG (*n* = 1,331)**	**TMVr + PCI (*n* = 29)**	***P*** **-value**	**SMVr + CABG (*n* = 133)**	**TMVr + PCI (*n* = 29)**	***P*** **-value**
Age, years (mean ± SD)	68.25 ± 8.46	72.38 ± 10.93	0.078	71.93 ± 8.22	72.07 ± 10.44	0.199
Female sex, *n* (%)	411 (30.9)	15 (51.7)	0.017	68 (51.1)	15 (51.7)	0.911
**Race**			0.851			0.593
White	1003 (77.9)	25 (86.2)		104 (78.8)	26 (89.7)	
African American	98 (7.6)	1 (3.4)		15 (11.4)	1 (3.4)	
Hispanic	101 (7.8)	2 (6.9)		4 (3)	2 (6.9)	
Asian/Pacific Islander	39 (3.0)	1 (3.4)		6 (3.7)	1 (3.4)	
Native American	7 (0.5)	0 (0)		3 (2.3)	1 (3.4)	
Other races	39 (3.0)	0 (0)		1 (0.75)	0 (0)	
**Comorbidities and medical history**						
Coronary heart disease	1331 (100)	29 (100)				
Hypertension, *n* (%)	473 (35.3)	3 (10.3)	0.05	14 (10.5)	3 (10.3)	0.932
Type 2 Diabetes mellitus, *n* (%)	512 (38.5)	8 (27.6)	0.233	62 (46.6)	13 (44.8)	0.146
Myocardial infarction, *n* (%)	1094 (82.2)	24 (82.8)	0.210	85 (73.9)	20 (69)	0.527
Heart failure, *n* (%)	836 (62.8)	28 (96.6)	<0.01	128 (96.2)	28 (96.6)	0.911
Cerebral infarction, *n* (%)	43 (3.2)	2 (6.9)	0.275	5 (3.8)	2 (6.9)	0.478
Liver disease, *n* (%)	65 (4.9)	2 (6.9)	0.620	15 (11.3)	3 (10.3)	0.840
Renal failure, *n* (%)	574 (43.1)	21 (72.4)	0.002	95 (71.4)	22 (75.9)	0.834
Peripheral vascular disease, *n* (%)	56 (4.2)	0 (0)	0.259	10 (7.5)	1 (3.4)	0.121
Chronic obstructive pulmonary disease, *n* (%)	314 (23.6)	7 (24.1)	0.945	41 (30.8)	7 (24.1)	0.416
Deficiency anemia, *n* (%)	56 (4.2)	0 (0)	0.259	7 (5.3)	1 (3.4)	0.199
Coagulopathy, *n* (%)	101 (7.6)	0 (0)	0.123	17 (12.8)	1 (3.4)	0.096
Obesity, *n* (%)	250 (18.8)	6 (20.7)	0.795	29 (21.8)	6 (20.7)	0.828
Alcohol use, %	43 (3.2)	0 (0)	0.325	3 (2.3)	1 (3.4)	0.406
Tobacco abuse, *n* (%)	433 (32.5)	7 (24.1)	0.339	30 (22.6)	7 (24.1)	0.927
Permanent pacemaker implantation	52 (3.9)	1 (3.4)	0.900	5 (3.8)	1 (3.4)	0.911
ICD implantation	25 (1.9)	3 (10.3)	0.001	4 (3.0)	3 (10.3)	0.088
**Primary payer, *n* (%)**			0.400			0.298
Medicare	829 (62.3)	23 (79.3)		104 (78.2)	20 (69)	
Medicaid	94 (7.1)	0 (0)		9 (6.8)	1 (3.4)	
Private insurance	343 (25.8)	5 (17.2)		34 (25.6)	6 (20.7)	
Other	64 (4.8)	1 (3.4)		9 (6.8)	2 (6.8)	

TMVr indicates transcatheter mitral valve repair; SMVR indicates surgical mitral valve repair; CABG indicates coronary artery bypass grafting; PCI indicates percutaneous coronary stent implantation.

## Results

### Characteristics of study participants selected from the NIS database

Between January 2016 and December 2018, a total of 9,036 patients who underwent SMVr and 3,785 patients who underwent TMVr were identified. After elimination, we finally selected 1,331 patients who underwent SMVr + CABG procedures and 29 patients who underwent TMVr + PCI procedures (Figure 1 and Table 1). Patients who underwent TMVr + PCI procedures were older compared to those who underwent SMVr + CABG procedures (72.38 years vs. 68.25 years, *P* = 0.078) (Table 1). Both cohorts included predominantly White patients (77.9% SMVr + CABG vs. 86.2% TMVr + PCI) (Table 1). The use of SMVr + CABG and TMVr + PCI was similar among Hispanic patients (7.8% vs. 6.9%) (Table 1). Compared to patients who received SMVr + CABG, those who received TMVr + PCI had higher proportions of female participants (51.7% vs. 20.9%, *P* = 0.017) and had a higher prevalence of heart failure (96.6% vs. 62.8%, *P* < 0.001), chronic renal failure (72.4% vs. 43.1%, *P* = 0.001), and ICD implantation (10.3% vs. 1.9%, *P* = 0.001), but SMVr + CABG had a higher prevalence rate of hypertension (35.3% vs. 10.3%, *P* = 0.05) (Table 1).

### Clinical outcomes in study cohort

To determine whether SMVr + CABG in patients with IMR leads to a higher risk of in-hospital mortality, periprocedural complications, and resource use, PSM was applied to reduce the bias due to confounding variables (Tables 1, 2). The results demonstrated that the in-hospital mortality was higher in the TMVr + PCI group compared with the SMVr + CABG group (11.8% vs.4.5%; *P* = 0.034, Table 2). The cost of care ($783548.80 ± 1743146.11 vs. $331846.523 ± 235718.27, *P* < 0.001) and the length of stay (17.9 ± 19.02 days vs. 15.44 ± 8.26 days, *P <* 0.001) were considerably higher for the TMVr + PCI group (Table 2). Patients who underwent SMVr + CABG were more likely to suffer from more blood transfusion (29.3% vs. 6.9%, *P* = 0.01; Table 2) post-procedural cardiogenic shock (11.3% vs. 0%, *P* = 0.044; Table 2).

**TABLE 2:** Clinical outcomes in patients who underwent SMVr + CABG and TMVr + PCI (2016–2018).

	Unmatched groups			Propensity-matched groups		
**Variable**	**SMVr + CABG (*n* = 1,329)**	**TMVr + PCI (*n* = 29)**	***P*** **-value**	**SMVr + CABG (*n* = 133)**	**TMVr + PCI (*n* = 29)**	***P*** **-value**
In-hospital mortality, *n* (%)	66 (5.0)	4 (13.8)	0.042	6 (4.5)	4 (13.8)	0.034
Length of hospital stay, days	12.23 ± 8.397	17.28 ± 19.309	<0.001	15.44 ± 8.26	17.9 ± 19.02	<0.001
Total charges, US$	294891.003 ± 232632.009	796410.72 ± 1772279.528	<0.001	331846.523 ± 235718.27	783548.80 ± 1743146.11	0.001
**Cardiac complications**						
Post-procedural cardiac tamponade, *n* (%)	11 (0.8)	0 (0)	0.623	3 (2.3)	0 (0)	0.406
Post-procedural cardiogenic shock, *n* (%)	61 (4.6)	0 (0)	0.238	15 (11.3)	0 (0)	0.044
Post-procedural cardiac arrest, *n* (%)	49 (3.7)	1 (3.4)	0.947	7 (5.3)	2 (6.7)	0.761
IABP, *n* (%)	182 (13.7)	7 (24.1)	0.107	27 (20.3)	8 (2.8)	0.443
ECMO, *n* (%)	14 (1.1)	0 (0)	0.579	1 (0.8)	0 (0)	0.634
Post-procedural pericardial complications, *n* (%)	52 (3.9)	0 (0)	0.278	8 (6.0)	1 (3.4)	0.561
**Respiratory complications**						
Post-procedural respiratory failure, *n* (%)	98 (7.4)	3 (10.3)	0.545	16 (12.0)	4 (13.3)	0.844
Post-procedural respiratory complications, *n* (%)	117 (8.8)	3 (10.3)	0.770	17 (12.8)	4 (13.3)	0.935
Post-procedural mechanical ventilation use, *n* (%)	203 (15.3)	6 (20.7)	0.422	35 (26.3)	7 (23.3)	0.736
**Other perioperative complications**						
Bleeding/hematoma post-procedure, *n* (%)	39 (2.9)	1 (3.4)	0.870	9 (6.8)	1 (3.4)	0.479
Post-procedural thrombosis due to cardiac prosthetic devices, *n* (%)	8 (0.6)	0 (0)	0.675	1 (0.8)	0 (0)	0.634
Post-procedural acute embolism and thrombosis, *n* (%)	22 (1.7)	2 (6.9)	0.034	2 (1.5)	2 (6.9)	0.099
Post-procedural blood transfusion, *n* (%)	304 (22.8)	2 (6.9)	0.042	39 (29.3)	2 (6.9)	0.01
Post-procedural acute kidney injury, *n* (%)	445 (33.4)	15 (51.7)	0.078	47 (35.3)	15 (51.7)	0.082
Fluid and electrolyte disorders, *n* (%)	578 (43.4)	10 (34.5)	0.336	72 (54.1)	11 (37.9)	0.084
Post-procedural cerebrovascular infarction or TIA, *n* (%)	19 (1.4)	0 (0)	0.517	2 (1.5)	0 (0)	0.499

TMVr indicates transcatheter mitral valve repair; SMVR indicates surgical mitral valve repair; CABG indicates coronary artery bypass grafting; PCI indicates percutaneous coronary stent implantation.

### Temporal trends

**FIGURE 2:**
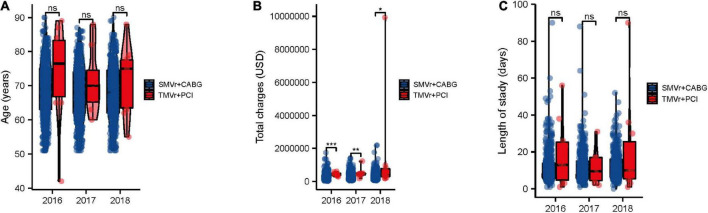
Trends in SMVr + CABG and TMVr + PCI from 2016 to 2018. Trends in age **(A)**, cost of stay **(B)**, and length of stay **(C)** of patients undergoing SMVr + CABG and TMVr + PCI from 2016 to 2018 in the National Inpatient Sample (NIS) database. TMVr indicates transcatheter mitral valve repair; SMVR indicates surgical mitral valve repair; CABG indicates coronary artery bypass grafting; PCI indicates percutaneous coronary stent implantation. *P < 0.05, **P < 0.01, ***P < 0.001.

Over the study period, patients in the SMVr + CABG group had the tendency of younger than those in the TMVr + PCI group, and the SMVr + CABG group had tendency of a lower total charge and a shorter length of stay when compared with the TMVr + PCI group (Figures 2A–C).

### Predictors of clinical outcomes

**FIGURE 3:**
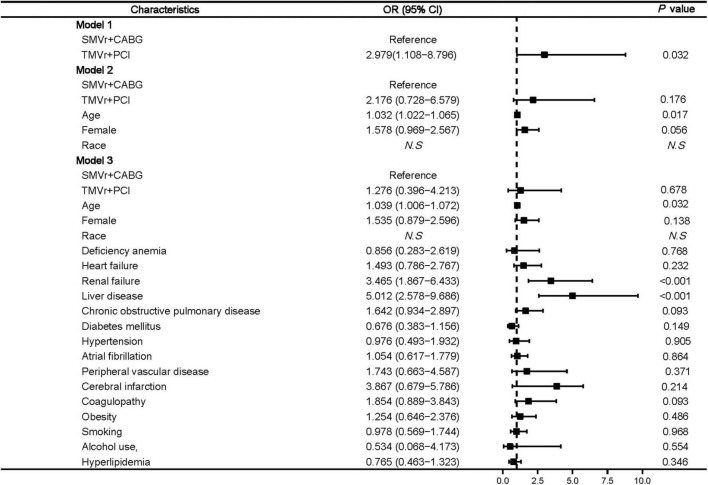
Predictors of mortality in mitral valve insufficiency patients undergoing SMVr + CABG or TMVr + PCI. TMVr indicates transcatheter mitral valve repair; SMVR indicates surgical mitral valve repair; CABG indicates coronary artery bypass grafting; PCI indicates percutaneous coronary stent implantation; model 1 indicates the univariate regression analysis; model 2 adjusted for SMVr + CABG, TMVr + PCI, age, female, race; model 3 adjusted for SMVr + CABG, TMVr + PCI, age, female, race, deficiency anemia, heart failure, renal failure, liver disease, chronic obstructive pulmonary disease, diabetes mellitus, hypertension, atrial fibrillation, peripheral vascular disease, cerebral infarction, coagulopathy, obesity, smoking, alcohol use, and hyperlipidemia.

Logistic regression showed that age (OR, 1.039 [95% CI, 1.006–1.072]; *P* = 0.032), renal failure (OR, 3.465 [95% CI, 1.867–6.433]; *P* < 0.001), and liver disease (OR, 5.012 [95% CI, 2.578–9.686]; *P* < 0.01) (Figure 3) were associated with higher mortality.

**FIGURE 4:**
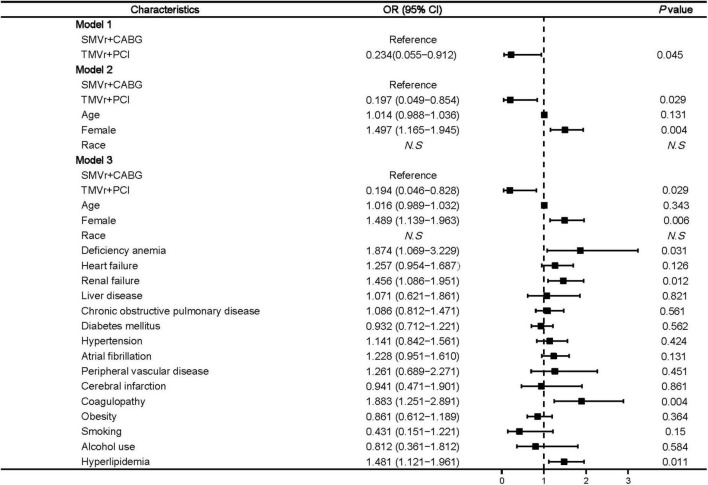
Predictors of post-procedural blood transfusion in mitral valve insufficiency patients undergoing SMVr + CABG or TMVr + PCI. TMVr indicates transcatheter mitral valve repair; SMVR indicates surgical mitral valve repair; CABG indicates coronary artery bypass grafting; PCI indicates percutaneous coronary stent implantation; model 1 indicates the univariate regression analysis; model 2 adjusted for SMVr + CABG, TMVr + PCI, age, female, race; model 3 adjusted for SMVr + CABG, TMVr + PCI, age, female, race, deficiency anemia, heart failure, renal failure, liver disease, chronic obstructive pulmonary disease, diabetes mellitus, hypertension, atrial fibrillation, peripheral vascular disease, cerebral infarction, coagulopathy, obesity, smoking, alcohol use, and hyperlipidemia.

Our results also suggested that SMVr + CABG was significantly related to blood transfusion (OR, 0.194 [95% CI, 0.046–0.828]; *P* = 0.029) (Figure 4). Factors associated with a higher rate of blood transfusion post-procedure included female sex (OR, 1.489 [95% CI, 1.139–1.963]; *P* = 0.006), renal failure (OR, 1.456 [95% CI, 1.086–1.951]; *P* = 0.012), and coagulopathy (OR, 1.883 [95% CI, 1.251–2.891]; *P* = 0.004) (Figure 4).

**FIGURE 5:**
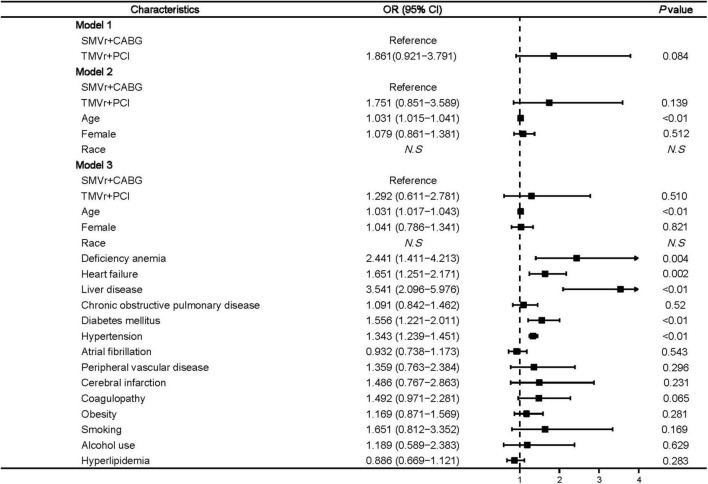
Predictors of post-procedural acute kidney injury in mitral valve insufficiency patients undergoing SMVr + CABG or TMVr + PCI. TMVr indicates transcatheter mitral valve repair; SMVR indicates surgical mitral valve repair; CABG indicates coronary artery bypass grafting; PCI indicates percutaneous coronary stent implantation; model 1 indicates the univariate regression analysis; model 2 adjusted for SMVr + CABG, TMVr + PCI, age, female, race; model 3 adjusted for SMVr + CABG, TMVr + PCI, age, female, race, deficiency anemia, heart failure, liver disease, chronic obstructive pulmonary disease, diabetes mellitus, hypertension, atrial fibrillation, peripheral vascular disease, cerebral infarction, coagulopathy, obesity, smoking, alcohol use, and hyperlipidemia.

Factors associated with a higher rate of post-procedural acute kidney injury included age (OR, 1.031 [95% CI, 1.017–1.043]; *P* < 0.01), deficiency anemia (OR, 2.441 [95% CI, 1.411–4.213]; *P* = 0.004), heart failure (OR, 1.651 [95% CI, 1.251–2.171]; *P* = 0.002), liver disease (OR, 3.541 [95% CI, 2.096–5.976]; *P* < 0.01), diabetes mellitus (OR, 1.556 [95% CI, 1.221–2.011]; *P* < 0.01), and hypertension (OR, 1.343 [95% CI, 1.239–1.451]; *P* < 0.01) (Figure 5).

## Discussion

The following main findings were reported in our contemporary real-world study of outcomes for SMVr + CABG vs. concurrent TMVr + PCI: (1) The length of stay in the hospital, medical cost, and in-hospital mortality were significantly higher for TMVr + PCI compared to SMVr + CABG; (2) TMVr + PCI was associated with improved perioperative complications compared with SMVr + CABG.

To date, there are very limited studies that have evaluated the efficacy and safety of SMVr for the treatment of patients with HF and FMR, and only several small observational studies have shown that SMVr improves LV functional status (10–12). SMVr and TMVr have been compared in several small observational studies, as well as in a subgroup of the EVEREST randomized trial for the treatment of patients with HF and FMR. Kortlandt and colleagues compared 365 FMR patients treated with TMVr to 95 patients treated with TMVR and showed that there was no significant difference in survival between the two groups at 3 years of follow-up (13). In the EVEREST trial subgroup of the 56 patients with FMR, the study compared the TMVr and SMVR for the 5 years of follow-up, and the results showed that there were no significant differences between TMVr and SMVr regarding the mortality, mitral valve surgery or reoperation, and 3+ or 4+ mitral regurgitation. Two other studies specifically compared SMVr using ring annuloplasty with TMVr in patients with unmatched FMR (14, 15). There was a single-center retrospective study including 76 patients treated with SMVr and 95 patients treated with TMVr; results showed that SMVr significantly reduced mitral regurgitation and mortality after 6 months of follow-up. Likewise, in another retrospective cohort study of 65 patients treated with SMVr and other 55 patients treated with TMVr, SMVr was founded to reduce mitral regurgitation more consistently and with more comparable mortality at a median 4 years of follow-up.

Most patients with moderate to severe IMR are primarily treated with GDMT, cardiac resynchronization therapy, and coronary artery revascularization for their underlying cardiomyopathy (8). The role of SMVr as the primary approach to ameliorating clinical outcomes of patients with FMR needs to be further determined (16). And according to a recent RCT study, using SMVr in combination with CABG for IMR treatment remains debatable for patients with moderate IMR (17). Although the benefits regarding the outcomes of perioperative complications are uncertain, the benefits seen in patients with remodeled ventricles and scar favor combined SMVr and CABG (18). Here, in our study, we demonstrated that TMVr + PCI was associated with higher resource use and in-hospital mortality, but associated with improved perioperative complications when compared with SMVr + CABG.

Recently, the effectiveness of TMVr in addition to GDMT compared with GDMT alone was investigated in the two RCT studies of MITRA-FR and COAPT (5, 19). Although the MITRA-FR results demonstrated neutral results without any benefit of TMVr (MitraClip) for the composite outcomes events of mortality and HF rehospitalization at 1 and 2 years of follow-up, the COAPT study displayed that TMVr (MitraClip) was favorable regarding cumulative HF rehospitalizations, as well as mortality at 2 and 3 years of follow-up (20). In addition, some of the studies have evaluated the efficacy and clinical outcomes of transcatheter TMVr (MitraClip) and SMVr among patients with secondary mitral regurgitation (21–23). However, there are very limited studies to compare the efficacy and clinical outcomes of TMVr + PCI and SMVr + CABG among patients with FMR.

In this study, our data suggested that the patients who underwent TMVr + PCI were accompanied by higher in-hospital mortality, post-procedural acute kidney injury, and more resources used, and multivariable analysis showed that TMVr + PCI is not associated with improved outcomes compared with SMVr + CABG. The reason for the more mortality in the TMVr + PCI group may be because there were more high risks patients in this group.

There are some limitations to this study because of the inherent weakness of the NIS database. First, NIS is a database based on administrative claims that use ICD codes for diagnosis, and that may lead to error or result in inaccuracy when we use the NIS samples to estimate the burden of comorbidities and complications. Second, NIS collects data on in-patient discharges, and each admission was registered as an independent event. Third, the long-term endpoints could not be evaluated in the NIS samples because the NIS database was not designed to follow-up the patients longitudinally, and for the patients in the TMVr + PCI group, the TMVr intervention for IMR may be too early, because there may have a reverse remodeling after PCI.

In conclusion, TMVr + PCI was associated with higher resource use and in-hospital mortality but with improved perioperative complications compared with SMVr + CABG. More clinical studies and RCTs are needed to compare TMVr + PCI vs. SMVr + CABG in patients with IMR.

## Data availability statement

The raw data supporting the conclusions of this article will be made available by the authors, without undue reservation.

## Ethics statement

Ethical approval was not provided for this study on human participants because the NIS database is publicly available, we do not need to get the approval of the institutional review board or the informed consent in our clinical study. The patients/participants provided their written informed consent to participate in this study.

## Author contributions

XW conceived the study and wrote the manuscript. XF provided the data and revised the manuscript. YM analysis the data and revised the manuscript. JL, TW, LZ, CL, and QC revised the manuscript and reviewed the results. GG and JW revised the manuscript and provided comments of this research. SP, ZL, and YZ revised the manuscript and provided guidance for this study.

## References

[1] Bartko PE Heitzinger G Pavo N Heitzinger M Spinka G Prausmüller S Burden, treatment use, and outcome of secondary mitral regurgitation across the spectrum of heart failure: observational cohort study. *BMJ.* (2021) 373:n1421. 10.1136/bmj.n1421 34193442 PMC8243241

[2] Otto CM Nishimura RA Bonow RO Carabello BA Erwin JP III Gentile F 2020 ACC/AHA guideline for the management of patients with valvular heart disease: a report of the American college of cardiology/American heart association joint committee on clinical practice guidelines. *Circulation.* (2021) 143:e72–227. 10.1161/cir.0000000000000923 33332150

[3] Grigioni F Enriquez-Sarano M Zehr KJ Bailey KR Tajik AJ. Ischemic mitral regurgitation: long-term outcome and prognostic implications with quantitative Doppler assessment. *Circulation.* (2001) 103:1759–64. 10.1161/01.cir.103.13.1759 11282907

[4] Jamieson WR Edwards FH Schwartz M Bero JW Clark RE Grover FL. Risk stratification for cardiac valve replacement. National cardiac surgery database. Database committee of the society of thoracic surgeons. *Ann Thorac Surg.* (1999) 67:943–51. 10.1016/s0003-4975(99)00175-7 10320233

[5] Stone GW Lindenfeld J Abraham WT Kar S Lim DS Mishell JM Transcatheter mitral-valve repair in patients with heart failure. *N Engl J Med.* (2018) 379:2307–18. 10.1056/NEJMoa1806640 30280640

[6] Feldman T Foster E Glower DD Kar S Rinaldi MJ Fail PS Percutaneous repair or surgery for mitral regurgitation. *N Engl J Med.* (2011) 364:1395–406. 10.1056/NEJMoa1009355 21463154

[7] Glower DD Kar S Trento A Lim DS Bajwa T Quesada R Percutaneous mitral valve repair for mitral regurgitation in high-risk patients: results of the EVEREST II study. *J Am Coll Cardiol.* (2014) 64:172–81. 10.1016/j.jacc.2013.12.062 25011722

[8] Otto CM Nishimura RA Bonow RO Carabello BA Erwin JP III Gentile F 2020 ACC/AHA guideline for the management of patients with valvular heart disease: executive summary: a report of the American college of cardiology/American heart association joint committee on clinical practice guidelines. *Circulation.* (2021) 143:e35–71. 10.1161/cir.0000000000000932 33332149

[9] Khera R Angraal S Couch T Welsh JW Nallamothu BK Girotra S Adherence to methodological standards in research using the national inpatient sample. *JAMA.* (2017) 318:2011–8. 10.1001/jama.2017.17653 29183077 PMC5742631

[10] Bach DS Bolling SF. Early improvement in congestive heart failure after correction of secondary mitral regurgitation in end-stage cardiomyopathy. *Am Heart J.* (1995) 129:1165–70. 10.1016/0002-8703(95)90399-2 7754949

[11] Bolling SF Pagani FD Deeb GM Bach DS. Intermediate-term outcome of mitral reconstruction in cardiomyopathy. *J Thorac Cardiovasc Surg.* (1998) 115:381–6. 10.1016/s0022-5223(98)70282-x 9475533

[12] Chen FY Adams DH Aranki SF Collins JJ Jr. Couper GS Rizzo RJ Mitral valve repair in cardiomyopathy. *Circulation.* (1998) 98(Suppl. 19):II124–7. 9852893

[13] Kortlandt F Velu J Schurer R Van den Branden B Bouma B Kelder J Impact of mitral valve treatment choice on mortality according to aetiology. *EuroIntervention.* (2019) 14:1733–9. 10.4244/eij-d-18-00874 30585781

[14] Conradi L Treede H Rudolph V Graumüller P Lubos E Baldus S Surgical or percutaneous mitral valve repair for secondary mitral regurgitation: comparison of patient characteristics and clinical outcomes. *Eur J Cardiothorac Surg.* (2013) 44:490–6. 10.1093/ejcts/ezt036 23401496

[15] De Bonis M Taramasso M Lapenna E Denti P La Canna G Buzzatti N MitraClip therapy and surgical edge-to-edge repair in patients with severe left ventricular dysfunction and secondary mitral regurgitation: mid-term results of a single-centre experience†. *Eur J Cardiothorac Surg.* (2016) 49:255–62. 10.1093/ejcts/ezv043 25669650

[16] Wu AH Aaronson KD Bolling SF Pagani FD Welch K Koelling TM. Impact of mitral valve annuloplasty on mortality risk in patients with mitral regurgitation and left ventricular systolic dysfunction. *J Am Coll Cardiol.* (2005) 45:381–7. 10.1016/j.jacc.2004.09.073 15680716

[17] Goldstein D Moskowitz AJ Gelijns AC Ailawadi G Parides MK Perrault LP Two-year outcomes of surgical treatment of severe ischemic mitral regurgitation. *N Engl J Med.* (2016) 374:344–53. 10.1056/NEJMoa1512913 26550689 PMC4908819

[18] Velazquez EJ Lee KL Jones RH Al-Khalidi HR Hill JA Panza JA Coronary-artery bypass surgery in patients with ischemic cardiomyopathy. *N Engl J Med.* (2016) 374:1511–20. 10.1056/NEJMoa1602001 27040723 PMC4938005

[19] Obadia JF Messika-Zeitoun D Leurent G Iung B Bonnet G Piriou N Percutaneous repair or medical treatment for secondary mitral regurgitation. *N Engl J Med.* (2018) 379:2297–306. 10.1056/NEJMoa1805374 30145927

[20] Mack MJ Lindenfeld J Abraham WT Kar S Lim DS Mishell JM 3-year outcomes of transcatheter mitral valve repair in patients with heart failure. *J Am Coll Cardiol.* (2021) 77:1029–40. 10.1016/j.jacc.2020.12.047 33632476

[21] Okuno T Praz F Kassar M Biaggi P Mihalj M Külling M Surgical versus transcatheter repair for secondary mitral regurgitation: a propensity score-matched cohorts comparison. *J Thorac Cardiovasc Surg.* (2021). 10.1016/j.jtcvs.2021.07.029 [Epub ahead of print]. 34446288

[22] Koschutnik M Dannenberg V Donà C Nitsche C Kammerlander AA Koschatko S Transcatheter versus surgical valve repair in patients with severe mitral regurgitation. *J Pers Med.* (2022) 12:90. 10.3390/jpm12010090 35055405 PMC8779938

[23] Nappi F Antoniou GA Nenna A Michler R Benedetto U vtaar Singh SSA Treatment options for ischemic mitral regurgitation: a meta-analysis. *J Thorac Cardiovasc Surg.* (2022) 163:607–22.e14. 10.1016/j.jtcvs.2020.05.041 32713629

