# Supplementary material for: Outcomes of transcatheter edge-to-edge mitral valve repair with percutaneous coronary intervention vs. surgical mitral valve repair with coronary artery bypass grafting
Source: Front Cardiovasc Med. 2022 Dec 21;9:953875. doi: 10.3389/fcvm.2022.953875 (PMC9810628; doi:10.3389/fcvm.2022.953875)
Supplement: Supplementary file 1 [file Table_1.docx]

**Table S1. Criteria to determine presence of diseases in NIS database.**

| **Diseases** | **Diagnoses code** |
| --- | --- |
| Ischemic cardiomyopathy | ICD-10-CM diagnoses code I255 |
| Coagulopathy | ICD-10-CM diagnoses code D684, D68311, D6851, D68312, D6861, D689, D6832, D682, D681, D6862, D68318, D6859, D688, D6869, D6852, D680 |
| Obesity | ICD-10-CM diagnoses code E669, E668, E6609, E661, E6601, E662 |
| Hypertension | ICD-10-CM diagnoses code I10, I152, I151, I158, I150, I159 |
| Coronary artery disease | ICD-10-CM diagnoses code I25111, I25118, I25119, I25110, I2510, I252, I255, I256, I25812, I25810, I25811, I2582, I2584, I2583, I2589, I259 |
| Atrial fibrillation | ICD-10-CM diagnoses code I482, I4820, I4811, I4819, I480, I4821, I481, I4891 |
| Diabetes mellitus | ICD-10-CM diagnoses code E10.x-E13.x, |
| Peripheral vascular disease | ICD-10-CM diagnoses code I7389, I739, I70201, I70209, I70503, I70502, I70508, I70501, I70509, I70603, I70602, I70608, I70601, I70609, I70703, I70702, I70708, I70701, I70709, I70303, I70302, I70308, I70301, I70309 |
| Hypercholesteremia | ICD-10-CM diagnoses code E7801, E780, E7800 |
| Alcohol use | ICD-10-CM diagnoses code F10180, F1014, F10150, F10151, F10159, F10181, F10182, F10121, F10120, F10129, F10188, F1019, F10131, F10132, F10130, F10139, F1011, F1010, F10280, F1024, F1026, F1027, F10250, F10251, F10259, F10281, F10282, F10221, F10220, F10229, F10288, F1029, F10231, F10232, F10230, F10239, F1021, F1020, F10980, F1094, F1096, F1097, F10950, F10951, F10959, F10981, F10982, F10921, F10920, F10929, F10988, F1099, F10931, F10932, F10930, F10939 |
| Tobacco abuse | ICD-10-CM diagnoses code Z716, Z720, F17221, F17220, F17228, F17229, F17223, F17291, F17290, F17298, F17299, F17293 |
| Cardiac shock | ICD-10-CM diagnoses code R570 |
| Sudden cardiac arrest | ICD-10-CM diagnoses code Z8674 |
| Ventilator use | ICD-10-PCS codes 5A1935Z, 5A1945Z, 5A1955Z |
| IABP | ICD-10-PCS codes 5A02210 |
| ECMO | ICD-10-PCS codes 5A15223, 5A1522F, 5A1522G, 5A1522H |
| Fluid and electrolyte disorders | ICD-10-CM diagnoses code E872, E873, E8770, E875, E870, E876, E871, E874, E878, E8779, E8771 |
| Chronic liver disease | ICD-10-CM diagnoses code K7211, K7210' |
| Peripheral vascular disease | ICD-10-CM diagnoses code I70201, I70209, I70503, I70502, I70508, I70501, I70509, I70603, I70602, I70608, I70601, I70609, I70703, I70702, I70708, I70701, I70709, I70303, I70302, I70308, I70301, I70309, I7389, I739 |
| Chronic obstructive pulmonary disease | ICD-10-CM diagnoses code J441, J440, J449, J418, J411, J410, J432, J439, J438, J431, J430, J40, J42 |
| Coronary artery disease | ICD-10-CM diagnoses code I25111, I25118, I25119, I25110, I2510, I252, I255, I256, I25812, I25810, I25811, I2582, I2584, I2583, I2589, I259 |
| Cerebral hemorrhage | ICD-10-CM diagnoses code I613, I614, I611, I610, I612, I615, I616, I619, I618, I6201, I6203, I621, I629, I6202, I6200 |
| Cerebral infarction | ICD-10-CM diagnoses code I636, I6312, I63423, I63133, I63443, I63413, I63433, I63113, I63422, I63132, I63442, I63412, I63432, I63112, I6349, I6319, I63421, I63131, I63441, I63411, I63431, I63111, I63429, I63139, I63449, I6340, I63419, I63439, I6310, I63119, I6302, I63323, I63033, I63343, I63313, I63333, I63013, I63322, I63032, I63342, I63312, I63332, I63012, I6339, I6309, I63321, I63031, I63341, I63311, I63331, I63011, I63329, I63039, I63349, I6330, I63319, I63339, I6300, I63019, I6322, I63523, I63233, I63543, I63513, I63533, I63213, I63522, I63232, I63542, I63512, I63532, I63212, I6359, I6329, I63521, I63231, I63541, I63511, I63531, I63211, I63529, I63239, I63549, I6350, I63519, I63539, I6320, I63219, I639, I638, I6389, I6381 |
| Renal failure | ICD-10-CM diagnoses code N171, N172, N170, N179, N178, N19, N990, N178 |
| Heart failure | ICD-10-CM diagnoses code I5041, I5031, I5021, I5042, I5032, I5022, I5043, I5033, I5023, I5041, I5031, I5043, I5033, I5042, I5032, I5040, I5030, I5041, I5043, I5023, I5021, I5042, I5022, I5020 |
| Hemopericardium | ICD-10-CM diagnoses code I312 |
| Cardiac tamponade | ICD-10-CM diagnoses code I314 |
| Respiratory failure after processure | ICD-10-CM diagnoses code J95822, J95821 |
| Respiratory complications | ICD-10-CM diagnoses code, J9562, J9561, J9572, J9571, J9588, J95861, J95860, J95831, J95830, J95863, J95862, J9589, J95821, J95822 |
| Mechanical ventilation use | ICD-10-CM diagnoses code Z9911 |
| Blood transfusion | ICD-10-PCS codes 30243N0, 30243N1, 30243P0, 30243P1, 30243H0, 30243H1, 30240N0, 30240N1, 30240P0, 30240P1, 30240H0, 30240H1, 30230H0, 30230H1, 30230N0, 30230N1, 30230P0, 30230P1, 30233N0, 30233N1, 30233P0, 30233P1 |
| Acute kidney injury | ICD-10-CM diagnoses code N171, N172, N170, N179, N178, N19, N990, R34, R944 |
| Postprocedural cerebrovascular infarction | ICD-10-CM diagnoses code I97810, I97811, I97820, I97821, G458, G459 |
| Pericardial complications | ICD-10-CM diagnoses code I312, I314, I301, I300, I308, I309, I310, I311, I313, I319 |
| Bleeding/hematoma post-procedure | ICD-10-CM diagnoses code I97630, I97631, I97638, I97621, I9762, I97610, I97611, I97618, I97620, I97411, I97410, I97418, I9742 |
| Thrombosis due to cardiac prosthetic devices, implants and grafts, initial encounter | ICD-10-CM diagnoses code T82867A, T82868A |
| Post procedure Cardiogenic Shock | ICD-10-CM diagnoses code T81.11 |
| Complication with Post-Procedure Stroke or TIA | ICD-10-CM diagnoses code I97810, I97811, I97820, I97821, G458, G459 |
| Bleeding/Hematoma Post-Procedure | ICD-10-CM diagnoses code I97621, I97630, I97631, I97638, I97410, I97411, I97418, I9742, I97410, I97411, I97418, I9742, I97610, I97611, I97618, I97620. |
| Cardiac Device Thrombus or Device Embolization | ICD-10-CM diagnoses code T82.867A, T81.817A |
| Acute embolism and thrombosis | ICD-10-CM diagnoses code I82492, I82493, I82491, I82499, I82463, I82413, I82423, I82462, I82412, I82422, I82452, I82432, I82442, I82453, I82433, I82461, I82411, I82421, I82451, I82431, I82441 |
| Mitral insufficiency | ICD-10-CM diagnoses code I340, I051, I341 |
| Mitral stenosis | ICD-10-CM diagnoses code I342, I050 |
| Mitral stenosis with insufficiency | ICD-10-CM diagnoses code I052 |
| Aortic insufficiency | ICD-10-CM diagnoses code I351, I061 |
| Aortic stenosis | ICD-10-CM diagnoses code I350, I060 |
| Aortic stenosis with insufficiency | ICD-10-CM diagnoses code I352, I062 |
| Tricuspid insufficiency | ICD-10-CM diagnoses code I361, I071 |
| Tricuspid stenosis | ICD-10-CM diagnoses code I360, I070 |
| tricuspid stenosis with insufficiency | ICD-10-CM diagnoses code I362, I072 |
| Pulmonary valve insufficiency | ICD-10-CM diagnoses code I371 |
| Pulmonary valve stenosis | ICD-10-CM diagnoses code I370 |
| Pulmonary valve stenosis with insufficiency | ICD-10-CM diagnoses code I372 |
| PCI | ICD-10-PCS codes 02703ZZ, 02704ZZ, 02713ZZ, 02714ZZ, 02723ZZ, 02724ZZ, 02733ZZ, 02734ZZ, 02Q03ZZ, 02Q04ZZ, 02Q13ZZ, 02Q14ZZ, 02Q23ZZ, 02Q24ZZ, 02Q33ZZ, 02Q34ZZ, 0270346, 027034Z, 0270356, 027035Z, 0270366, 027036Z |
| CABG | ICD-10-PCS codes 0210083, 0210088, 0210089, 021008C, 021008F, 021008W, 0210093, 0210098, 0210099, 021009C, 021009F, 021009W, 02100A3, 02100A8, 02100A9, 02100AC, 02100AF, 02100AW, 02100J3, 02100J8, 02100J9, 02100JC, 02100JF, 02100JW, 02100K3 |
| TMVR | ICD-10-PCS codes 02UG37E, 02UG37Z, 02UG38E, 02UG38Z, 02UG3JE, 02UG3JZ, 02UG3KE, 02UG3KZ, 02UG47E, 02UG47Z, 02UG48E, 02UG48Z, 02UG4JE, 02UG4JZ, 02UG4KE, 02UG4KZ |
| TMVr | ICD-10-PCS codes '02QG3ZE, 02QG3ZZ, 02QG4ZE, 02QG4ZZ, 02RG37H, 02RG37Z, 02RG38H, 02RG38Z, 02RG3JH, 02RG3JZ, 02RG3KH, 02RG3KZ, 02RG47Z, 02RG48Z, 02RG4JZ, 02RG4KZ, 02UG37E, 02UG37Z, 02UG38E, 02UG38Z, 02UG3JE, 02UG3JZ, 02UG3KE, 02UG3KZ, 02UG47E, 02UG47Z, 02UG48E, 02UG48Z, 02UG4JE, 02UG4JZ, 02UG4KE, 02UG4KZ |
| OMVR | ICD-10-PCS codes 02QG0ZE, 02QG0ZZ, 02UG07E, 02UG07Z, 02UG08E, 02UG08Z, 02UG0JE, 02UG0JZ, 02UG0KE, 02UG0KZ' |
| OMVr | ICD-10-PCS codes 02QG0ZE, 02QG0ZZ |
| TTVr | ICD-10-PCS codes 02QJ3ZG, 02QJ3ZZ, 02QJ3ZG |
| STVr | ICD-10-PCS codes 02QJ0ZG, 02QJ0ZZ |
| STVR | ICD-10-PCS codes 02RJ07Z, 02RJ0JZ |

TMVr indicates transcatheter mitral valve repair; TMVR, transcatheter mitral valve replacement; OMVr, open approach mitral valve repair; OMVR, open approach mitral valve replacement; STVr indicates surgical tricuspid valve repair; STVR indicates surgical tricuspid valve replacement; TTVr indicates transcatheter tricuspid valve repair; ECMO, extracorporeal membrane oxygenation; IABP, intra-aortic balloon pump.
